# Implications of endotracheal tube biofilm in ventilator-associated pneumonia response: a state of concept

**DOI:** 10.1186/cc11357

**Published:** 2012-05-23

**Authors:** Sara Gil-Perotin, Paula Ramirez, Veronica Marti, Jose Miguel Sahuquillo, Eva Gonzalez, Isabel Calleja, Rosario Menendez, Juan Bonastre

**Affiliations:** 1 Servicio de Medicina Intensiva, Hospital Universitario y Politécnico La Fe, Bulevar Sur s/n, 46026, Valencia, Spain; 2 Servicio de Microbiología, Hospital Universitario y Politécnico La Fe, Bulevar Sur s/n, 46026, Valencia, Spain; 3 Servicio de Neumología, Hospital Universitario y Politécnico La Fe, Bulevar Sur s/n, 46026, Valencia, Spain

## Abstract

**Introduction:**

Biofilm in endotracheal tubes (ETT) of ventilated patients has been suggested to play a role in the development of ventilator-associated pneumonia (VAP). Our purpose was to analyze the formation of ETT biofilm and its implication in the response and relapse of VAP.

**Methods:**

We performed a prospective, observational study in a medical intensive care unit. Patients mechanically ventilated for more than 24 hours were consecutively included. We obtained surveillance endotracheal aspirates (ETA) twice weekly and, at extubation, ETTs were processed for microbiological assessment and scanning electron microscopy.

**Results:**

Eighty-seven percent of the patients were colonized based on ETA cultures. Biofilm was found in 95% of the ETTs. In 56% of the cases, the same microorganism grew in ETA and biofilm. In both samples the most frequent bacteria isolated were *Acinetobacter baumannii *and *Pseudomonas aeruginosa*. Nineteen percent of the patients developed VAP (*N *= 14), and etiology was predicted by ETA in 100% of the cases. Despite appropriate antibiotic treatment, bacteria involved in VAP were found in biofilm (50%). In this situation, microbial persistence and impaired response to treatment (treatment failure and relapse) were more frequent (100% vs 29%, *P *= 0.021; 57% vs 14%, *P *= 0.133).

**Conclusions:**

Airway bacterial colonization and biofilm formation on ETTs are early and frequent events in ventilated patients. There is microbiological continuity between airway colonization, biofilm formation and VAP development. Biofilm stands as a pathogenic mechanism for microbial persistence, and impaired response to treatment in VAP.

## Introduction

The presence of an endotracheal tube (ETT) in ventilated patients impairs mucocilliary clearance and disrupts the cough reflex, thus promoting the accumulation of tracheobronchial secretions and increasing the risk of pneumonia [1]. In addition, the insertion of an ETT could produce injury and inoculate endogenous oropharyngeal bacteria in the low airway tract [2]. Finally, formation of biofilm on the surface of ETT is an almost universal phenomenon and it has been related to the pathogenesis of ventilator-associated pneumonia (VAP). Hence, due to the role of ETTs in the pathophysiological development of VAP, some authors suggest that it should be renamed ETT-associated pneumonia [3].

Microorganisms attach to synthetic surfaces, multiply and develop biofilms characterized by the generation of an extracellular polymeric substance or matrix that has been well documented with scanning electron microscopy (SEM) studies [4-7]. Biofilms have great importance for public health because of their role in certain infectious diseases and their role in a variety of device-related infections [8-14]. In those device-related infections, biofilms have been also involved in bacterial antibiotic resistance that depends on multicellular strategies [15,16]. This resistance implies, in most of the cases, the necessity of device withdrawal in order to achieve clinical and microbiological cure.

Bacterial biofilm has been observed universally on the surface of endotracheal tubes in mechanically ventilated patients [17-20]. Some data show a good concordance between bacterial colonization of the airway and microbial findings in the biofilm [19]. Even the same microorganisms causing VAP could be found in the ETT biofilm leading to the potential implication of biofilm in the genesis of VAP [19,21-23]. In fact, a recent study has demonstrated the efficacy of a novel silver-coated ETT in decreasing the incidence of microbiologically confirmed VAP [24], although no statistically significant between-group differences were observed in duration of intubation, intensive care unit stay, and hospital stay or mortality.

Microbial persistence in respiratory airways of pathogens implicated in VAP, even with an appropriate antibiotic therapy, has been related to the lack of response to treatment and to the relapse of VAP [25,26]. Bacterial survival in ETT biofilm can promote VAP microbial persistence and consequently affect patient prognosis. However, no attempt has been performed in order to assess the relationship among biofilm, microbial persistence and outcome of the VAP episode.

The main purpose of the current investigation was to investigate the involvement of ETT biofilm in VAP pathogenesis, response to treatment and relapse. Simultaneously, we assessed more precisely the sequence of airway bacterial colonization and the prevalence of ETT biofilm in mechanically ventilated patients.

## Materials and methods

### Patient data

In a seven-month period, we prospectively recruited in our study all the patients consecutively admitted to our Medical Intensive Care Unit that required mechanical ventilation for at least 24 hours. We did not include the same patient in case of re-intubation. The protocol was reviewed and approved by the Institutional Review Board, and the patients (or their relatives) provided informed consent to participate in the study. The written consent included the permission to collect and publish (anonymously) personal data concerning the patients.

The following data were recorded in a standardized form: age and sex of the patient, Acute Physiology and Chronic Health Evaluation (APACHE) II score [27], cause of mechanical ventilation, duration of ventilation, maneuvers to prevent VAP, the occurrence or not of nosocomial pneumonia, together with data of pathogen isolated in respiratory samples and/or ETT biofilm, antibiotic therapy and sensitivity patterns.

Maneuvers to prevent VAP, such as elevation of the head of the bed and subglottic aspiration, were performed in all our patients. Selective digestive decontamination protocol consisted of intravenous cefotaxime 1 g/8 h for three days plus nasogastric administration of 0.5 g of 2% antibiotic gel with 100 mg polymyxin E+ 80 mg tobramycin + 500 mg amphotericin B every 6 h while duration of mechanical ventilation.

### Definitions

VAP was defined as previously reported [28]. The diagnosis of VAP in our study was: a) clinical: new or progressive lobar infiltrates > 48 hours after intubation, and two or more of the following minor criteria (fever, leukocytosis/leukopenia, and purulent respiratory secretions) and was microbiologically confirmed [29]. Microbiology of VAP was determined by mini-bronchial-alveolar lavage (miniBAL) obtained after clinical suspicion and confirmed VAP when yielded > 1,000 UFC/ml. Empirical therapy and the management of patients were based in ATS/IDSA guidelines [30]. We considered as early-onset VAP those episodes that were initiated four days or less upon intubation [31-33]. Appropriate antimicrobial therapy was defined as coverage of all pathogens isolated by the antimicrobial therapy administered at the onset of VAP determined by the sensitivity pattern in the anti-biogram [34].

Microbial persistence was defined as the persistence of the causative microorganism of the VAP episode in at least two successive respiratory samples, despite 72 hours of proper antibiotic therapy irrespective of colony counts [35].

VAP relapse was defined as reported [25]: (a) occurrence at least 72 h after clinical resolution; (b) positive bronchoscopic quantitative culture for previously isolated strain; (c) evidence for a new infiltrate on the chest X-ray; (d) two of the following: fever > 38°C; white blood cell (WBC) count > 10,000/mm^3^; or purulent respiratory secretions; and (e) absence of evidence of a new extrapulmonary source of infection. The definition of treatment failure included at least one of the following 72 hours after the initiation of treatment: (a) failure to improve the PaO2/FiO2 ratio or need of intubation because of pneumonia; (b) persistence of fever (> 38°C) or hypothermia (< 35°C) and purulent respiratory secretions; (c) worsening of pulmonary infiltrates (> 50%); (d) occurrence of septic shock or multiple organ dysfunction not present at the onset of pneumonia [25,36,37].

Clinical resolution was defined as stated elsewhere [38]: including: (1) röentgenographic improvement, (2) normothermia, (3) WBC ≥3,000/mm^3 ^or WBC ≤12,000/mm^3^, (4) completion of a course of antibiotic therapy.

### Respiratory samples

We performed surveillance sampling of endotracheal aspirates (ETA) at the time of intubation and twice a week with analysis in the Microbiology laboratory. If VAP was suspected, we obtained a mini-bronchial-alveolar lavage (miniBAL) before antibiotic administration (when possible) that was quantitatively cultured.

### Processing of ETT

After extubation (due to clinical improvement, change in the ETT for technical reasons, or death), we collected the ETTs, avoiding contamination other than from oropharyngeal flora. After rinsing with sterile saline buffer to eliminate the excess mucus, a 1-cm-long cross-sectional segment of the distal ETT (27 to 28 cm from the ETT connector piece) [39] was divided into two portions for both electron microscopy and bacterial cultures. Biofilm was scraped from the inner surface of the ETT and homogenized by vortex mixing, being cultured using standard techniques. For scanning electron microscopy (SEM), the section of the ETT was fixed in a mixture of 4% paraformaldehyde-2,5% glutaraldehyde in 0,1 M phosphate buffer, for one hour at room temperature. Samples were washed and dehydrated with crescent ethanol concentrations, dried with liquid CO_2_, sputter-coated, and examined under a Hitachi (S-4100) microscope (Tokyo, Japan) (SCSIE-University of Valencia, Spain). We then analyzed low magnification images (1,000×) to quantify biofilm extension, and high magnification images (15,000×) to identify attached microorganisms.

### Statistical analyzes

Results were expressed as mean ± SEM, or median (interquartile range) for continuous variables and the number (percentage) for dichotomous variables. Risk factors for biofilm formation and VAP outcomes were analyzed by univariate analysis, using the chi-square test/ANOVA for dichotomous variables and the Mann-Whitney U test for continuous variables. All statistical tests were two-tailed, and *P *< 0.05 was considered significant. We performed all analyses using the SPSS 16.0 (SPSS Inc, Chicago, IL, USA).

## Results

During the study period we collected data from 81 patients that required invasive mechanical ventilation. We finally included in our analysis 75 patients. Missed patients occurred due to two reasons: a) loss of the ETT after extubation (*n *= 2) and b) biofilm deterioration during fixation protocol (*n *= 4). The clinical features of these patients, as well as the reasons for mechanical ventilation are shown in Table 1.

**Table 1:** Baseline characteristics of patients

Variable^a^	*n *= 81
APACHE II, mean ± SEM	21 ± 0.7
Age, mean ± SEM	59 ± 1.8
Gender female, N/%	27/33%
Previous immunosuppression^b^, N/%	19/24%
Previous antibiotics, N/%	62/77%
SDD, N/%	30/37%
Diabetes mellitus, N/%	19/24%
Causes of intubation	
- Acute respiratory failure, N/%	31/38%
- Acute heart failure, N/%	10/12%
- Coma GCS < 7 to 8, N/%	40/49%

^a ^Indicates status at time of intubation ^b ^Active onco-hematologic disease, solid organ transplantation, hematopoietic stem cell transplantation

### Surveillance cultures

Bacterial growth in surveillance cultures was documented in 65 patients (87%). From the total ETA sampled (*n *= 252), 151 (60%) grew positive for microorganisms (most polymicrobial). The mean time from intubation to positivity of surveillance cultures was 2.1 ± 0.4 days. As regards bacterial growth, *Acinetobacter baumannii *grew in 20 patients (32%), *Pseudomonas aeruginosa *grew in 14 patients (22%) (all *A. baumannii *and *P. aeruginosa *were multidrug-resistant strains). Coagulase-negative staphylococcus (CNS) was isolated in 13 (20%), and 10 patients (15%) had positivity for *Staphylococcus aureus *(including both resistant and susceptible strains to methicilin). *Candida albicans *was isolated in 29 patients (45%), while other species of *Candida *grew in 17 patients (26%) (Table 2).

**Table 2:** Bacterial isolation in surveillance endotracheal aspirates

Microorganism	ETA n, %	Days until ETA+ (mean ± SEM)	ETA-ETT match (n, %)
Colonized patients	65, 87%	2.1 ± 0.4	36, 56%
*Acinetobacter baumannii*	20, 32%	7.8 ± 1.6	12, 60%
*Pseudomonas aeruginosa*	14, 22%	5.4 ± 2.1	9, 64%
*Cocci (SCN, Streptococcus spp)*	13, 20%	5 ± 0.9	4, 31%
*Staphylococcus aureus (MSSA,MRSA)*	10, 15%	2.2 ± 0.6	6, 60%
*Candida albicans*	29, 45%	2 ± 0.6	6, 21%
*Candida no albicans*	17, 26%	3.2 ± 0.5	1, 6%

### Biofilm formation

**Figure 1:**
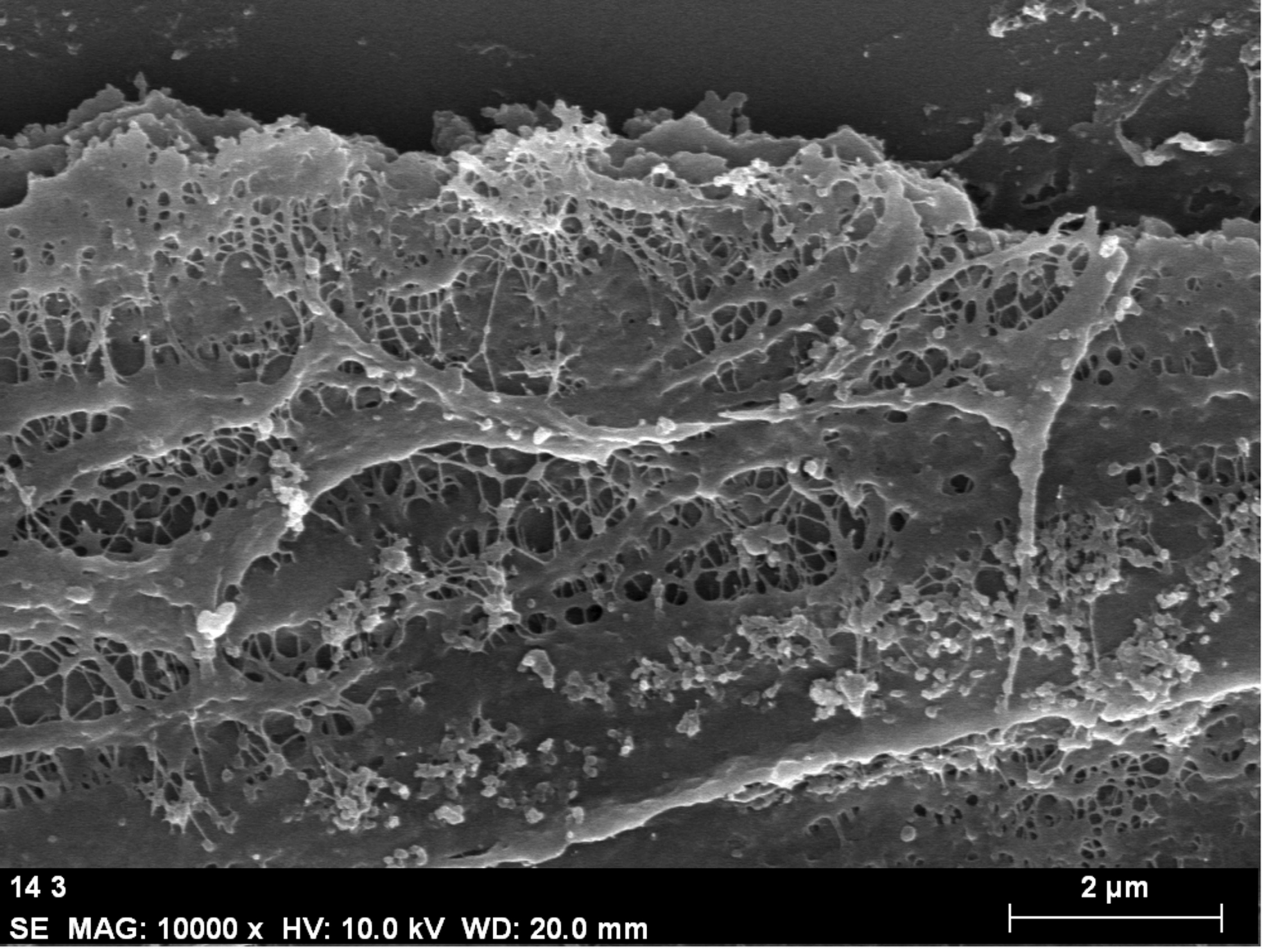
**Scanning electron microscopy micrographs of biofilm in the endotracheal tubes**. Biofilm at low magnification is composed of a matrix that attaches on the surface of the ETT. Scale bar: 2 μm.

**Figure 2:**
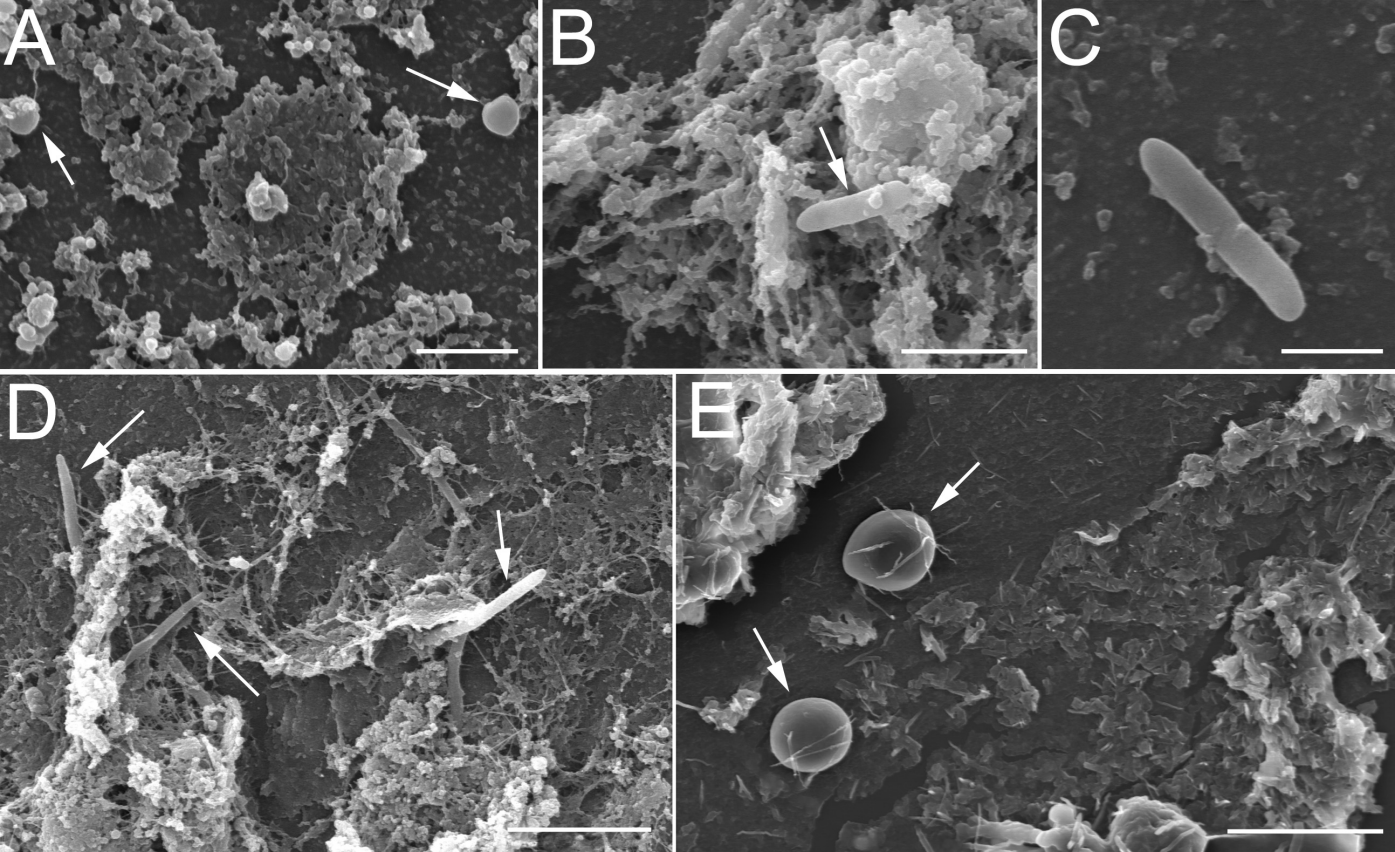
**Identification of microorganisms on the surface of biofilm**. In certain cases we could identify microorganisms immersed in the biofilm matrix. **A) **Cocci, scale bar: 2 μm, **B-D) **Bacilli, scale bar: 4 μm, 2 μm and 5 μm, respectively, and **E) **yeast. Scale bar: 10 μm.

Biofilm was present in 71 (95%) ETTs. The extension and morphology of biofilm was variable, from ETTs with scarce biofilm (13, 18%), with dispersed clusters (26, 37%) or with confluent abundant biofilm matrices extended in the inner surface of the tubes (29, 41%) as depicted in Figure 1. Presence of biofilm was noted from as early as 24 hours after intubation, being discovered incipient patches of biofilm in ETTs (9, 13%). Identifiable microorganisms occasionally remained on the surface of the biofilm (Figure 2). Biofilm formation was not associated with the duration of stay of the ETT, administration of selective digestive decontamination (SDD), systemic antibiotics or immunosuppression.

Seventy-three (97%) ETTs were microbiologically assessed, and bacterial growth was documented in 60 of them (83%). The most frequent isolations were *A. baumannii *(17, 28%), *P. aeruginosa *(14, 23%), and CNS (13, 22%). *C*. *albicans *was isolated in six ETTs (10%) and *C. glabrata *in one patient (2%). We then compared cultures of the last ETA obtained before extubation and ETT biofilm. From the previously colonized patients (*n *= 65), the same microorganisms could be found in the ETT biofilm in 36 patients (56%). If we only considered those patients colonized by *A. baumannii *and *P. aeruginosa*, the percentage of concordance achieved 69%. Mismatch between ETA and biofilm (*n *= 37) was due to the absence of microbial isolation in the ETT in 10 cases (27%). In those patients with different pathogens in ETA and ETT, the most frequently involved microorganisms were *Candida *spp. (22 of the 27 cases). From the 46 patients colonized by *Candida *spp., only in 7 cases, *Candida *could be found in the ETT biofilm.

### Ventilator-associated pneumonia

Fourteen (19%) patients developed 17 episodes of VAP during their stay in ICU (including a 2^nd ^episode of VAP and 2 relapses). We only analyzed the first episode of VAP in every patient (*n *= 14). Early-onset pneumonia (≤4 days of intubation) (*n *= 3) was caused by *Haemophilus influenzae*, *Enterobacter cloacae *and *Streptococcus *spp. Microorganisms involved in late-onset pneumonia (*n *= 11) were *A*. *baumannii *(6, 54%), *P. aeruginosa *(3, 27%), methicilin-resistant *Staphylococcus aureus *(1, 9%), and CNS (1, 9%) (Table 3). Days of intubation (*P *= 0.05) and previous airway colonization by *P. aeurginosa *and *A. baumannii *(odds ratio (OR), 10.2; 95% CI, 2.11 to 49.3, *P *= 0.005 and OR: 4.9, 1.18 to 20.3; *P *= 0.03 respectively) were associated with the development of late-onset VAP. Age, gender, APACHE II score at admission, immunosuppression, diabetes, cause of ICU admission, cause of intubation, antibiotic treatment previous to intubation or biofilm extension on ETT did not influence VAP development.

**Table 3:** Ventilator-associated pneumonia aetiology, microbial persistence, and treatment failure

Patient	Causal pathogen	Early onset	Bacterial survival on ETT	Appropriate treatment	Persistence	Non-response	Relapse
*5*	*CNS*	No	No	Yes	Yes	No	No
*9*	*A. baumannii^a^*	No	Yes	Yes	Yes	Yes	No
*10*	*Streptococcus spp*.	Yes	No	Yes	No	No	No
*41*	*A. baumannii*	No	No	Yes	No	No	No
*48*	*MRSA*	No	Yes	Yes	Yes	No	No
*50*	*P. aeruginosa*	No	Yes	Yes	Yes	Yes	Yes
*51*	*E. cloacae*	Yes	No	Yes	No	No	No
*63*	*A. baumannii*	No	No	Yes	Yes	Yes	No
*67*	*H. influenzae*	Yes	No	Yes	No	No	No
*76*	*P.aeruginosa*	No	No	Yes	No	No	No
*80*	*A. baumannii*	No	Yes	Yes	Yes	No	No
*81*	*P. aeruginosa*	No	Yes	No	Yes	No	No
*83*	*A. baumannii*	No	Yes	Yes	Yes	Yes	No
*91*	*A. baumannii*	No	Yes	Yes	Yes	Yes	Yes

*^a ^P. aeruginosa *nosocomial pneumonia as cause of ICU admission

In all cases (*n *= 14), the surveillance ETA cultures predicted the etiology and antibiotic sensibility patterns in both early and late onset VAP, even 15 days before the VAP onset (median of 5 days [4-11]). Appropriate antibiotic therapy was prescribed in 13 cases (93%). In 50% of the cases (*n *= 7) the bacteria involved in VAP survived to an adequate antibiotic treatment and could be found in ETT biofilm (this percentage raised to 70% when *A. baumannii *and *P. aeruginosa *were the causal microorganisms in VAP).

### Relationship between biofilm, microbial persistence, non-responders and VAP relapse

Microbial persistence was detected in 9 (64%) patients with VAP. The bacteria most frequently implicated in microbial persistence were *A. baumannii *and *P. aeruginosa *(seven of the nine cases). Bacterial survival on biofilm was associated to microbial persistence (100% vs 29%; *P *= 0.021).

**Figure 3:**
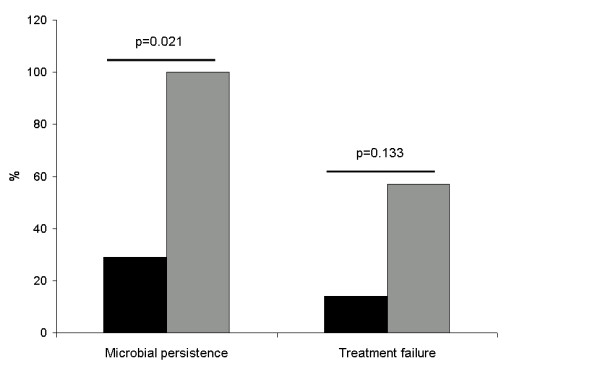
**Relationship between biofilm, microbial persistence and treatment failure**. Bar graph representative of the percentage of cases in which there was (gray) or not (black) bacterial survival on ETT biofilm despite appropriate treatment. Microbial persistence in respiratory samples and treatment failure were more frequent when bacterial growth was documented in ETT.

Treatment failure occurred in five patients (36%) and two patients (14%) had a VAP relapse (both relapse episodes occurred in already non-responding patients). All treatment failure and relapse episodes were related to *A. baumannii *and *P. aeruginosa*. Although not statistically significant, treatment failure was more frequent (57% vs 14%; *P *= 0.133) when the causal bacteria remained in ETT biofilm despite appropriate treatment (Figure 3).

## Discussion

The main findings of our study are the high prevalence of biofilm in ETT from mechanically ventilated patients, the microbial link between airway colonization, biofilm formation and VAP development, and the potential implication of bacterial survival on biofilm in VAP outcome.

Other authors have previously shown a high prevalence of biofilm on ETT, even at short permanence times [19]. We confirmed these results and were able to assess these observations in the case of multi-resistant gram-negative bacteria. *A. baumannii *and *P. aeruginosa *were the most prevalent bacteria in our study. *P. aeruginosa *has been involved in chronic respiratory infections and biofilm formation seems to be an important pathogenic factor in these patients. Likewise, environmental resistances of *A. baumannii *strains seem to be directly related to its capacity to form biofilm, as has been recently shown [37]. To the knowledge of the authors, this is the first time that biofilm formation by *A. baumannii *has been widely analyzed in ETT from mechanically ventilated patients. Duration of ventilation or the use of local or systemic antibiotics seemed to not influence biofilm formation in our study. Similar to us, Adair *et al. *found that selective digestive decontamination was ineffective in preventing biofilm formation on ETTs [40]. Other potential preventive measures, such as inhaled antibiotics or the use of special biomaterials on ETTs, were not fulfilled in this study [39,41].

Airway colonization by nosocomial bacteria is a common phenomenon and many investigations recognize a direct relationship between colonization and nosocomial pneumonia [42]. A total of 87% of our patients were colonized, most frequently by *A. baumannii *and *P. aeruginosa *(45%). In more than half of the patients (56%), the same bacteria could be found in ETA and ETT biofilm, 69% in the case of Gram-negative bacteria. Mismatch between ETA and ETT biofilm (different bacteria) was mainly due to *Candida *spp. isolation in ETA, and not in ETT. Preference of *Candida *spp. attaching mucosa rather than prosthesis has been shown in previous studies [43].

Despite the high prevalence of airway colonization and biofilm on ETT, only 14 patients developed pneumonia in our study group. Therefore, biofilm formation and airway colonization were necessary but not sufficient for VAP development. Among the known VAP risk factors, we found that days of mechanical ventilation and airway colonization by *A. baumannii *and *P. aeruginosa *increased the risk for late onset VAP [44,45]. In accordance with some of the studies performed on this topic, we found a high concordance between bacteria colonizing the airway and subsequently causing VAP [46-50].

Antibiotic resistance of bacteria embedded on biofilm has been studied with regard to difficult-to-treat device-related infections [12]. Because of bacterial survival, most of the current guidelines recommend device withdrawal when possible [51]. Treatment failure in VAP can occur despite the absence of an identifiable cause, that is, inappropriate treatment, concomitant infection, superinfection or non-infectious causes [37]. As in other device-related infections, bacterial survival on ETT biofilm could explain this lack of response to antibiotic treatment. In our study, bacteria causing VAP could be found in ETT biofilm in half of the cases, despite an appropriate antibiotic treatment. Even when our sample size prevents us from achieving forceful conclusions, we have observed a worse outcome of the VAP episode in those ETTs acting as a bacterial reservoir. Although more studies should prove our findings, it seems reasonable to hypothesize the potential benefit of a biofilm directed intervention in the setting of a VAP episode.

Even when our sample is one of the largest in the literature concerning biofilm formation on ETTs, the low number of patients with VAP is inadequate to achieve statistical forcefulness. However, the relationship between bacterial persistence and worse outcomes in VAP is stated, and becomes a relevant and novel association. We could describe in great detail the presence and characteristics of biofilm in ETT from mechanically ventilated patients but some of the interesting associations with VAP outcome observed need to be confirmed in future studies. Another potential limitation of our study is the use of qualitative surveillance cultures. The value of qualitative versus quantitative ETA in order to study airway colonization is a topic for discussion. The studies regarding this subject show heterogeneous results. While some authors argue against the diagnostic value of qualitative ETA due to oropharyngeal and high airway contamination, they could not prove a better diagnostic performance with quantitative ETA [52]. A work by Aydogdu *et al. *showed an advantage of qualitative compared to quantitative surveillance ETA in predicting VAP aetiology [53]. Even more, the necessity of quantitative cultures has been recently questioned. Niederman *et al. *identified methodological limitations of quantitative cultures that could cause both false-positive and false-negative results. According to this review, accuracy of clinical scores associated with Gram stain of lower respiratory tract secretions were not improved by the use of quantitative cultures [54].

## Conclusions

In summary, our study supports the idea of a dynamic relationship among airway colonization, biofilm and VAP development. The idea of bacterial survival on ETT biofilm as a pathogenic mechanism for microbial persistence and impaired response to antibiotic therapy highlights the importance of discovering new strategies focused in the removal of biofilm from the ETT.

## Key messages

• Biofilm is a frequent and precocious finding in mechanically-ventilated patients

• A high percentage of patients have their airway colonized based on ETA surveillance cultures, mostly by *A. baumanii *and *P. aeruginosa*.

• VAP etiology was predicted by ETA in all cases with a median of five days between the first positive ETA and VAP occurrence.

• Microbial persistence and impaired response to treatment (treatment failure and relapse) were more frequent when multidrug-resistant microorganisms were present in ETT biofilm.

• Biofilm stands as a pathogenic mechanism for microbial persistence and impaired response to treatment in VAP.

## Abbreviations

APACHE II: Acute Physiology and Chronic Health Evaluation II; CNS: coagulase-negative staphylococcus; ETA: endotracheal aspirates; ETT: endotracheal tube; ICU: intensive care unit; miniBAL: mini bronchial-alveolar lavage; SDD: selective digestive decontamination; SEM: scanning electron microscopy; VAP: ventilator-associated pneumonia; WBC: white blood cell.

## Competing interests

The authors declare that they have no competing interests.

## Authors' contributions

SGP and PR conceived and designed the study, carried out the processing of the ETT for electron microscopy, observed the biofilm in the microscope, performed the statistical analysis, and drafted the manuscript. VM and IC helped with sample collection and with processing the ETT. JMS and EG performed microbiological analysis of the respiratory samples and ETT biofilm. RM and JM helped with the design and coordination of the study and with drafting the manuscript. All authors read and approved the final manuscript.
